# A multilevel approach for promoting physical activity in rural communities: a cluster randomized controlled trial

**DOI:** 10.1186/s12889-019-6443-8

**Published:** 2019-01-30

**Authors:** Alan M. Beck, Amy A. Eyler, J. Aaron Hipp, Abby C. King, Rachel G. Tabak, Yan Yan, Rodrigo S. Reis, Dixie D. Duncan, Amanda S. Gilbert, Natalicio H. Serrano, Ross C. Brownson

**Affiliations:** 1 Prevention Research Center in St. Louis, Brown School, Washington University in St. Louis, One Brookings Drive, Campus Box 1196, St. Louis, MO 63130 USA; 2 North Carolina State University, Raleigh, USA; 3 Department of Health Research and Policy (Epidemiology) and the Stanford Prevention Research Center (Department of Medicine), Stanford University School of Medicine, Stanford, CA USA; 4 Department of Surgery, (Division of Public Health Sciences) and Alvin J. Siteman Cancer Center, Washington University in St. Louis, St. Louis, USA; 5 Graduate Program in Urban Management (PPGTU), School of Architecture and Design, Pontifical Catholic University of Paraná (PUCPR), Curitiba, Brazil

**Keywords:** Physical activity, Rural residents, Cancer prevention

## Abstract

**Background:**

Physical activity (PA) has demonstrated a decreased risk in various cancers and other chronic diseases; however, rural residents are less likely to attain recommended levels of PA compared to urban and suburban counterparts. Given rural residents make up 15% of the United States population, there is a need for novel approaches to increase PA among this population. The goal of the present study is to investigate the effectiveness of a multilevel intervention to increase PA rates among rural residents.

**Methods/design:**

Guided by an ecological framework, a group-randomized design will be used to evaluate the effects of a three-level intervention for increasing PA among adult residents residing in 6 rural communities (*n* = 600) along with 6 control communities (n = 600). The intervention includes components at the individual (short message service [SMS] text messages), interpersonal (social support in walking groups), and community levels (events at existing trails). Innovative methods to encourage participation will be employed as well as a focus on life priorities (family, recreation, hobbies) other than health. Aim 1 includes a literature review and key informant interviews to determine the local contexts for intervention adaptation. Aim 2 will employ a set of interventions at the individual, interpersonal, and community-levels to evaluate their impact on moderate-to-vigorous PA as measured by self-reported (telephone survey) and objectively assessed (accelerometry) measures. These data are supplemented by location based on Global Positioning System and community audits, which provide information on recreational amenities, programs/policies, and street segments.

**Discussion:**

This study is among the first of its kind to test a multilevel intervention in a rural setting, address life priorities that compliment health outcomes, and examine moderation between behavioral interventions and the natural environments where people are physically active. Our results will influence the field by enhancing the ability to scale-up innovative, PA interventions with the potential to reach high-risk, rural populations.

**Trial registration:**

Clinical Trials NCT03683173, September 25, 2018.

## Background

Cancer is a major public health threat, accounting for 25% of all deaths, and is the second most common cause of death in the United States [1, 2]. Moreover, up to 50% of cancer-related deaths are preventable through evidence-based lifestyle modifications, such as increasing physical activity (PA) levels, enhancing nutrition, and reducing obesity rates [3–5]. Among major cancer risk factors, physical inactivity is estimated to cause 12.4% of breast cancer and 12.0% of colon cancer [6].

Across the US, approximately 21% of the adult population achieves the recommended 150 min of PA per week [7]. PA rates are disproportionately lower in rural areas, with rural residents least likely to meet PA recommendations compared to urban and suburban residents [8]. Further, rates of PA are lowest in smaller rural communities (i.e., populations under 10,000 persons) [9]. Accordingly, decreased PA rates among rural residents elicits a higher risk for cancer and other chronic diseases, as compared to urban/suburban residents [10, 11]. Rural residents make up 15% of the US population [12]; therefore, it is imperative to determine effective interventions for increasing PA in order to decrease risk of chronic diseases.

There is little evidence for increasing PA in rural areas at the population level. The present study aims to utilize feedback from community members and key stakeholders to deliver an effective and tailored multilevel intervention. The multilevel intervention will consist of events at local walking trails, formation of walking groups, and text messaging. This manuscript describes a two-group randomized controlled trial to investigate the effectiveness of a multilevel intervention designed to increase PA rates among rural populations.

### Intervention background and development

In the past decade, research on the connection between the built environment (BE) and PA has grown considerably [13–15]. It remains unclear if findings from urban areas can be generalized to rural communities due to differences in culture, population densities, physical environment, and other contextual factors [16, 17]. In a review, safety, aesthetics, and the existence of parks, walking trails and recreation centers were positively associated with PA in rural residents [18]. Yet, even with the existence of these amenities, the use of trails remains low (regular use from 15 to 33%) [19–21].

Changing the BE (e.g., building walking trails) is likely a necessary but not sufficient approach for increasing rates of PA—that is, communication/promotional efforts are warranted [22–24]. PA and obesity intervention success has been shown to be moderated by proximity, access, and use of natural environments [25–27]. For example, Epstein et al. [25] found greater park area near one’s home was associated with decreased body mass index. Merom et al. [27] demonstrated the success of a promotional campaign encouraging use of a rail-trail was moderated by proximity to the trail. PA in outdoor settings, as compared to indoor settings, is associated with a number of co-benefits including feelings of revitalization, engagement, enjoyment, and decreases in anger, tension, and depression [28]. In addition, growing evidence suggests a moderating (interactive) effect between proximity to parks/green space and physical activity interventions [29, 30]. The lone review focusing on built environment effects in rural settings concluded research is limited by methods and external validity, and consists mainly of cross-sectional studies in middle-aged adults [18]. While increasing evidence shows the importance of the built environments (e.g., mixed use development, presence of parks) in supporting PA [13, 18, 31–35], few studies in rural communities are available [16].

Walking groups provide participants with social support to increase exercise adherence [36, 37]. Further, walking groups have been found to be advantageous for enhancing health [38], increasing PA [39], and increasing interconnectedness among neighbors [40]. Despite the potential benefits of walking groups, little is known about whether the results seen in urban/suburban populations generalize to rural counterparts. In a rural study, Brownson et al. [41] found a tendency of walking groups to impact PA; however, recommend a higher dose intervention to detect significant results.

Access to cell phones are nearly ubiquitous—in 2015, 92% of Americans owned a cell phone and coverage rates have increased every year since 2010 [42]. In 2014, 88% of rural residents owned a cell phone [43]. With worldwide availability and shrinking costs of mobile phones, more options are available for mobile health intervention delivery. Advice and support through short message service (SMS, or text messaging) has shown promise for improving adherence in diabetes self-management and weight loss [44–48]. However, relatively less SMS research has been aimed specifically at PA in underserved populations, particularly those living in rural communities.

Text messaging has excellent health promotion potential given mobile phones and text messaging are commonly used among the majority of US adults, including rural residents and those who have limited computer and reading literacy skills [49, 50]. In addition, multiple studies have shown the feasibility of reaching lower income, rural residents with simple cell phone-based communication channels such as text messaging [51–53]. While a few SMS PA programs are currently available, most lack formal evaluation and published evidence of effectiveness. Those SMS PA programs that have been scientifically tested, have not been assessed specifically among rural populations, who are at high risk for physical inactivity [9].

A recent study explored issues such as interests (e.g., volunteering); values (e.g., personal health); awareness, access, and use of local trails; cell phone use; and physical activity level using a telephone interview with 524 adults from eight towns in rural Missouri (i.e., towns with trails in the same region as the current study) [54]. In this study, individuals who reported doing some walking but did not meet PA recommendations were identified as a key group to target. Participants were asked, “what sort of events or activities could be held at the trail that might encourage you to visit the trail?” Many respondents mentioned community events, such as picnics, races or walking events, festivals, and kid-friendly activities. Others mentioned sports and having social support, such as group walks or walking partners. Working in the yard, bike riding, fishing, dancing, hiking and camping were done more often within the past 6 months among those who walk. Relationships with friends, personal health, finances/housing/standard of living, conditions at work/job satisfaction, being outdoors and in nature were rated as more important among those who use walking trails compared to those who did not.

The present study is based on research demonstrating: 1) the burden of physical inactivity is large, increasing, and disproportionately affects rural residents; 2) the pressing need for high-impact, scalable interventions; 3) new technologies (e.g., SMS) need to be tested in rural settings; and 4) participatory approaches supporting life priorities other than health show promise for increasing the effectiveness of PA interventions.

## Methods/design

This is a mixed methods study involving qualitative and quantitative components including, 1) a set of key informant interviews with local individuals to inform intervention activities, and 2) a cluster randomized controlled trial whereby six intervention and six control sites will be matched based upon population, availability of a walking trail, non-white population, and population below poverty. The study will take place in a 10-county region in rural southeast Missouri. Most researchers define rural at the county level or metropolitan level as nonmetropolitan areas with less than a 50,000 population [9, 55, 56]. Based on the US classification system [57], most communities in our study will be nonmicropolitan rural (population size < 9999) with a few micropolitan rural towns (population size 10,000 to 49,999). The intervention area has a poverty rate twice that of Missouri overall [58]. Based on pilot work, at least 39 walking trails have been built in the region [59]. The study was approved by the institutional review board of Washington University in St. Louis.

**Fig. 1:**
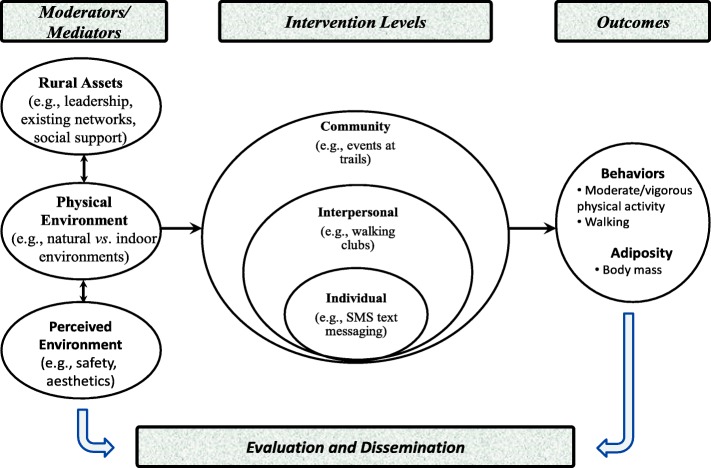
Framework for promoting physical activity in rural communities

An ecological (multilevel) framework will be used to organize the study. Several different ecological frameworks have been developed to help explain the complexities of health behavior [60–63]. Ecological models point to the importance of addressing problems at multiple levels, and the interaction and integration of factors within and across all levels. In this study, data will be collected and analyzed across three major levels: 1) community factors, 2) interpersonal factors, and 3) individual factors (see Fig. 1).

### Study aims and approaches

#### Aim 1: Inform intervention refinement and questionnaire development through the latest science and engagement with local residents and leaders

Involvement of stakeholders in designing, implementing, and evaluating projects leads to more relevant and effective approaches [64–68]. This study plans to assemble an advisory committee, which may be linked to coalition formation in some counties, to provide: 1) overall project guidance; 2) review of survey methods and instruments; 3) input on the interventions; 4) guidance on evaluation; 5) assistance in disseminating our findings; and 6) guidance on sustaining and scaling up findings. The committee will include academic members, leaders of professional organizations in public health from southeastern Missouri, and community members.

Public health researchers have long benefited from collecting community members perspectives concerning the problems they face in reaching behavioral health targets [64]. Community input can pave the way for more comprehensive solutions. Aim 1 participants include 60 adults sampled from three groups (20 per group): 1) trail users; 2) individuals who live within 2 km of the trails but do not use them regularly; and 3) agency partners. Key informant interviews will allow for the collection of information from a range of people who have first-hand knowledge of local resources, culture, and behaviors. Because key informants need to be knowledgeable about what is being studied, purposive sampling will be used to select participants (i.e., subjects are selected because of who they are and what they know, rather than by chance) [69]. The interviews will focus on three major areas: 1) perception of trails in the community; 2) barriers and enablers of trail use; and 3) ideas on activities that could be held at the trail to promote use by community members.

The development of the multilevel intervention will come directly from the communities via key informant interviews. The information gleaned from the interviews will allow for tailoring text messages, formation of walking groups, and creation of community events aimed at increasing awareness of local walking trails. The key informant interviews will allow us to engage with local stakeholders for collaboration and committee formation.

Within the first aim of the study is also the development of a locally appropriate telephone survey. We will use existing networks in the area to first conduct cognitive response testing (CRT) on a draft of the survey. CRT is a systematic method to understand a respondent’s thought process and how they arrive at a given answer [70]. The emphasis in CRT is on how the respondent comprehends a question and arrives at a response, not the actual response. We will pursue a probing technique whereby the interviewer will ask probing questions after the participant reads a given question and possible responses aloud. Feedback from CRT will lead to culturally and locally appropriate questions and response options for Phase 2 of the study. After CRT, we will conduct a pilot test of the survey with residents of the catchment area; however, the participants will be from nearby towns not included in Phase 2.

#### Aim 2: Test the independent and moderating effects of a multilevel intervention and the community environment on physical activity among rural residents

We will evaluate the effectiveness of a multilevel intervention on PA, behavioral, psychosocial, and anthropometric measures among rural adult residents. The target audience for our study includes adult residents in 12 communities (6 intervention, 6 control) in southeastern Missouri who are able to perform PA. Given the high rate of poverty in the region (with all counties significantly higher than the Missouri poverty rate of 15.5% [71], the study population will be lower income than the overall Missouri population. We will also sample a significant African American population (from 10 to 27%) in five of the 10 southeastern Missouri counties. To analyze the interventions’ impact on PA, we will collect pre, post 1-year, and post 2-year data regarding participants self-reported PA (a subset will also be assessed with objective accelerometry and GPS measures), height and weight, beliefs and attitudes related to PA, support for PA, and demographic variables.

### Participants/inclusion criteria

Aim 2 participants include 1200 (at baseline) adults who are: 1) 18–70 years of age; 2) able to be physically active [72, 73]; 3) reside in targeted communities with a walking trail (6 intervention, 6 control); and 4) willing to complete surveys at three time points: i) baseline, ii) one-year follow-up, and iii) two-years follow-up; however, participants are not required to have a SMS capable telephone. There will be a subset of the main cohort (*n* = 900) who agree to wear an accelerometer and global positioning system (GPS) device at the same intervals as the survey.

### Exclusion criteria

Participants will be considered ineligible if they do not provide consent, are unable to be physically active, or are unwilling or unable to complete surveys at three time points.

### Recruitment

**Fig. 2:**
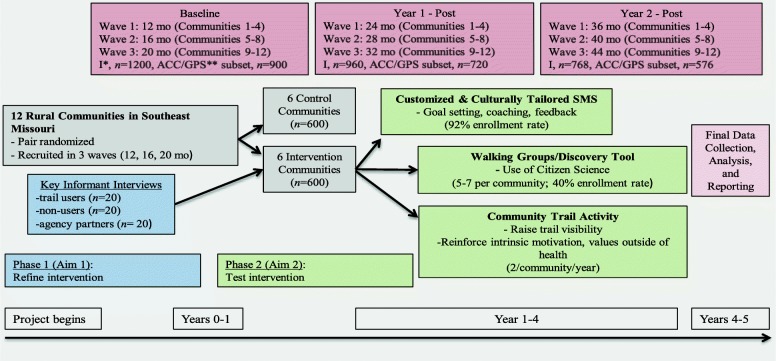
Study Schema. *I = Interview; **ACC = accelerometer; GPS = global positioning system

Intervention participants will be enrolled by active (e.g., phone calls, word of mouth, churches, businesses, and referrals) and passive (e.g., newspaper advertisements, social media, leaflet drops, and posters) strategies. A two-stage random digit dialing method will be used to select respondents in control communities, with sampling proportional to population size of each matched intervention community [74]. Within each control community, sampling will be stratified based on distance to the trail, gender, and age to ensure continuity between intervention and control communities on key variables likely to impact physical activity. Recruitment of participants will occur in three waves and will be facilitated by project staff and existing networks (see Fig. 2). Informed consent will be completed via a telephone script read to the participant.

The method by which an individual is recruited will be recorded and as follow-up data are analyzed, relative change in PA can be assessed within various methods of enrollment – allowing for generalizability according to reach (e.g., number participating in intervention activities) and impact (e.g., changes in PA behavior).

### Intervention

The *Heartland Moves* (HM) intervention will consist of three main aspects: 1) holding events at local walking trails to encourage attendance and increase visibility, 2) the formation of local walking groups or support and grow current walking groups, and 3) enrollment into a 24-week exercise SMS. We expect a dose-response relationship with the multilevel intervention whereby the more levels a given participant engages in, the higher the probability of increased PA. As noted in the inclusion criteria section, we do not require all participants to have the ability to receive text messages.

The HM walking groups and community events are informed by the Self-Determination Theory (SDT), which has emerged as an increasingly prevalent theory to explain physical activity behavior [75, 76]. A 2012 review of 66 empirical studies connecting SDT and PA found autonomy to be strongly correlated with PA [75]. Another review of three intervention trials found consistent support for using SDT in PA promotion [77]. Our use of SDT will focus on relatedness (social linkages in walking groups) and intrinsic motivation based on priorities other than health (local culture, hobbies, family).

The HM SMS intervention portion of our study is based on evidence-informed principles and strategies drawn primarily from Social Cognitive Theory (SCT), the Transtheoretical Model, and SDT. While many health interventions try to motivate behavior change to improve health, there are often other priorities besides health (physiological, safety, belonging, enjoyment) [78]. The intervention emphasizes individually tailored self-management skills, self-regulation skill building, personalized advice and feedback, social support, and user-driven choice/decision-making [79]. Both SCT and SDT have explanatory power for individual-level behavior change, and have intra-individual elements that are modifiable through communication and counseling [80–83]. Substantial evidence supports these theories, which have moderate effect sizes to improve PA [75, 77, 84, 85].

### Data collection

A telephone-based survey will collect demographic, behavioral, beliefs and attitudes toward PA, support for PA, and self-reported PA. Accelerometers and GPS devices will capture objective PA and location of PA (e.g., in neighborhood or at walking trails) from a subset of participants. Data collection will occur in waves and consist of baseline, 1-year, and 2-year follow-up (see Fig. 2). The principal investigator, project manager, and project statistician will conduct data monitoring. All adverse events will be reported immediately by the project manager to the institutions institutional review board.

### Measures

#### Primary outcome

Objectively assessed PA will be measured on a subset of the participants via 7-day accelerometry (Actigraph wGT3X-BT) and global positioning system (QStarz BT-Q1000XT) wear at baseline, 1-year, and 2-year.

#### Secondary outcomes

Self-reported PA will be measured with a modified Global Physical Activity Questionnaire (GPAQ) [86]. The GPAQ is a 16-item survey measuring vigorous and moderate PA levels related to work, travel, recreational activities, and sedentary behavior; however, we will measure sedentary behavior with the Marshall Sitting Questionnaire [87]. The Marshall Sitting Questionnaire is a 5-item survey measuring sedentary (sitting) behaviors related to travel, work, television watching, technology use (e.g., computer, tablet, phone), and leisure time on weekdays and weekends.

### Mediators, moderators, and covariates

#### Walking

Walking behaviors and preferences will be measured with a 15-item survey originally developed by Brownson et al. [88].

#### Physical activity support

Physical activity support will be measured with the 5-item Physical Activity Support Scale [89].

#### Perceived environment

Perceived environment will be measured via the Rural Active Living Perceived Environment Support Scale (RALPESS) [90], and a shortened version of the Neighborhood Environment Walkability Scale (NEWS) [91]. The RALPESS is a 33-item survey related to environmental support in indoor areas, outdoor areas, town center, schools, churches, and areas around one’s home. The NEWS is a 29-item survey related to places for walking and cycling, neighborhood surroundings, and neighborhood safety; however, we will use only the items related to neighborhood safety.

#### Self-efficacy for exercise

Self-efficacy for exercise will be measured with Bandura’s 9-item Exercise Self-Efficacy Scale [92, 93].

#### Physical activity stages of change

Participants will be grouped into one of five stages as it relates to PA (pre-contemplation, contemplation, preparation, action, and maintenance). One’s specific stage will be measured with the 4-item Physical Activity Stages of Change Questionnaire [94].

#### Interests and values

Participants will be asked questions related to their interests and values in order to determine methods to increase PA in other ways. The scale to be used will be the interests and values scale developed by Park et al. [54].

#### Demographic variables

Demographic information will be collected on all participants, including age, gender, marital status, number of children, educational attainment, race/ethnicity, employment status, and income.

#### Body mass index (BMI)

Height and weight will be collected via self-report. BMI is calculated as the ratio of weight in kilograms divided by the square of the height in meters.

### Randomization

Potential data collection sites will be paired based on population, income, and availability of walking trails. The study statistician will randomly select control and intervention sites based on proportion below the poverty line, proportion of non-white residents, and population.

#### Data management

Data will be double entered and checked for validity using range checks. Data will be stored in a confidential manner on protected servers. All confidential data collected will only be accessible to project staff. The dissemination plan for study results will include academic journals, conference presentations, funder reports, and results shared with the local population.

#### Qualitative analysis

Digital recordings of key informant interviews will be transcribed verbatim. Two project team members will analyze these transcripts. After reviewing the research questions [95, 96], the team members will read five of the transcripts using a first-draft of a code book. Each coder will be asked to systematically review the data and organize each of the statements into categories that summarize the concept or meaning articulated [97]. Once the first five transcripts are coded, they will be discussed in detail to ensure the accuracy of the codebook and inter-coder consistency. The codebook will be edited as needed prior to coding the remainder of the transcripts. All transcripts will be analyzed using NVIVO.12 [98]. Themes from the coded transcripts will be summarized and highlighted with exemplary quotes from participants. Data analysis may also include quantification or some other form of data aggregation, including the use of data matrices. Comparisons will be made to identify key differences in thematic issues between trail users, non-users, and agency leaders.

#### Sample size calculation

This study uses a paired, group-randomized design. By using matching criteria, we will balance potential community-level confounding factors (e.g., community size, poverty level) with a gain in the statistical power [99, 100]. Based on the literature, we estimated accelerometer-measured moderate-vigorous physical activity (MVPA) in the control group at 42.5 min per week (m/w) [9]. In the intervention group, we assume a 16% increase (42.5*1.16 = 49.3), based on a recent meta-analysis [101]. We used a much larger standard deviation (SD) of 16 rather than that reported (6.2) [9] to ensure a sufficient sample size both for testing the intervention effect and for testing the intervention-by-environment interaction (moderation) effect. We assumed intraclass correlation (ICC) estimates in the range of 0.01–0.03, based on our pilot studies and values of ICCs in the literature [102–111]. With 12 communities and an average of 48 study subjects in each (a total of 576 subjects for accelerometry), the study has power > 90% with 2-sided α = 5% to detect the mean difference in MVPA between the control (42.5) and intervention (49.3) groups, using Donner’s method [112]. For intervention-by-environment interaction, we assume in the natural environment, the intervention and control difference is 57 m/w versus 44 m/w, and in the indoor environment, the difference is 43 m/w versus 41 m/w. Estimates of interaction are based on our pilot work and existing studies [27, 30]. With a common SD in each of four subgroups = 10, we have 84% power to detect a significant interaction using factorial analysis of variance methods (F-test) adjusting for clustering (ICC = 0.03). Our simulation using two-level mixed effect models also indicates the power to detect the assumed interaction effect > 90%. Our sample size is determined for accelerometry subjects at the end of year 2. With a yearly follow-up rate of 80%, which is achievable given our previous work, [102–105, 111] we need to enroll 576/0.64 = 900 at baseline for accelerometry. For telephone survey subjects, we need to enroll 900/0.75 = 1200 at baseline.

#### Quantitative analysis

Both cluster-level and individual-level analyses will be performed in SAS. We designate Y_ijk_ as the year 2 PA level (minutes/week) for subject k with treatment j (j = 1 intervention, 0 control) in pair i(i = 1…6), and Y_ij_-bar is the mean in year 2 PA with treatment j in pair i, and d_i_ = (Y_i1_-bar – Y_i0_-bar), the difference in the mean between intervention and control communities in pair i. In addition, let X and Z be a vector of the relevant variables at the community and individual level, respectively.

For cluster (community) level analyses, we will follow Donner [112] for analysis of matched-pair quantitative data. Specifically, we consider d_i_ as the unit of analysis and will use the weighted paired *t*-test (with the cluster size as weights) if the cluster size varies across pairs. We will use the permutation test in which we compare the observed difference with the null distribution derived from the permutation procedure. Using a two-level model, multilevel analyses allow us to study individual outcomes affected by factors (main effect and cross-level interaction effects) at different levels. The basic model for the individual level analysis is: Y_jk_ = α_j_ + βI_j_ + γX_j_ + ηZ_jk_ + θ I_j_*E_jk_ + ε_jk_; where Y_jk_ is the change in PA for subject k in j^th^ community (j = 1,2…12), I = 1 if intervention and 0 otherwise, X = a vector of community level covariates, Z = a vector of individual level covariates (including E_jk_ individual level of activity environment), and I_j_*E_jk_ = intervention-by-environment interaction. The parameter α_j_ is the random intercept, β is the main effect for intervention, and θ is the interaction effect. In this model, β is the expected difference in the PA level between intervention and control among those whose PA is mainly indoor, and (θ + β) is the expected difference in the PA level between intervention and control among those whose PA is mainly in the natural environment. The intervention is at the community level, and the activity environment is classified at the individual level. Both main and interaction effects are adjusted for community level and individual level covariates. The primary analyses will be conducted at the end of 1 and 2-year follow-up. In these analyses, baseline PA level will be used as one of the covariates. In the primary analyses, all randomized subjects will be included in their original study group regardless of the final extent of compliance with the study protocol; that is, an “intent to treat” analysis.

## Discussion

This study will be among the first of its kind to systematically examine the effects of a multilevel intervention among rural residents living in areas of high poverty, a population at extremely high-risk of inactivity [9]. Among existing studies in rural settings, few have measured PA objectively. In addition, this study design allows for testing of the interaction between a multilevel intervention and the natural environment, which has rarely been achieved in rural settings.

A major strength of the study is the engagement of local residents in designing the approaches to be used. We are meeting the participants where they are to determine the best method by which to increase their PA. We are focusing on aspects of life, other than health, to positively impact the health of individuals. Engaging participants at every phase of the study could lead to comprehensive and creative solutions to increase PA among rural residents.

The dissemination potential of this study for these new technologies to address rural cancer risk is considerable [113]. Lastly, too often researchers have used the “push” model where new science is generated and pushed out to communities; this study will engage the community in intervention design and will focus on life priorities other than health as entry points or motivators for PA. This innovative approach will leave lasting positive effects within the communities served, as well as have potential to be scaled-up in the future.

### Potential limitations

Recruitment of a large number of study participants may be challenging. However, we believe we can recruit the proposed sample size in a timely fashion because of the large number of potentially eligible participants in the study regions and our success in recruiting and retaining individuals in similar studies [19, 114–117]. Local partners will be extremely valuable in these efforts. Participation in various intervention components is likely to vary by individual (e.g., some may be interested in text messaging but not in community events). Because the project is designed such that all participants do not need to participate in all intervention components and we will calculate a dose variable, we will account for this variable enrollment. The study cohort of high-risk individuals is at higher risk for loss to follow-up. We have accounted for these potential dropouts in our sample size calculations. In addition, we have a successful history of participant retention in other trials conducted in rural settings.

## Abbreviations

BE: Built environment
CRT: Cognitive response testing
GPAQ: Global physical activity questionnaire
GPS: Global positioning system
HM: Heartland moves
ICC: Intraclass correlation
m/w: Minutes per week
MVPA: Moderate-vigorous physical activity
NEWS: Neighborhood environment walkability scale
PA: Physical activity
RALPESS: Rural active living perceived environment support scale
SCT: Social-cognitive theory
SD: Standard deviation
SDT: Self-determination theory
SMS: Short message service
